# Severe Pulmonary Hypertension Caused by Smoldering Plasma Cell Myeloma: An Autopsy Case of POEMS Syndrome

**DOI:** 10.1155/2012/836893

**Published:** 2012-11-29

**Authors:** Katsuya Chinen, Yasunori Fujioka

**Affiliations:** ^1^Department of Pathology, Kyorin University School of Medicine, 6-20-2 Shinkawa, Tokyo, Mitaka 181-8611, Japan; ^2^Department of Pathology, Nerima General Hospital, 1-24-1 Asahigaoka, Tokyo, Nerima 176-8530, Japan

## Abstract

The POEMS syndrome (coined to refer to polyneuropathy, organomegaly, endocrinopathy, M protein, and skin changes) is a rare variant of plasma cell disorders with multiple systemic manifestations. Recently, pulmonary hypertension (PH) has become established as a complication, but pathological studies of this condition are scarce and the detailed pathogenesis remains to be elucidated. We present herein a case of a 49-year-old woman who was diagnosed as having idiopathic PH and was treated in accordance. However, she eventually died of respiratory failure and an autopsy revealed the presence of smoldering plasma cell myeloma and multiple organomegaly in addition to severe PH. The latter was attributed to stenosis and occlusion of the arterioles of the lungs due to marked plasma cell proliferation, quite different from the histology of idiopathic PH. From these findings, together with the clinical details, we concluded that the patient's PH was a complication of the POEMS syndrome. This case showed a unique pulmonary vascular pathology featuring plasma cell proliferation and it provides clues towards understanding the pathogenesis with this background.

## 1. Introduction

 The POEMS syndrome (coined to refer to polyneuropathy, organomegaly, endocrinopathy, M protein, and skin changes) is well known to be associated with a variety of clinical presentations, including pulmonary manifestations, renal impairment, hematological disorders, and extravascular volume overload [1–6]. The pathogenesis of POEMS syndrome is not well understood, but overproduction of proinflammatory cytokines, such as interleukin (IL)-1*β*, IL-6, tumor necrosis factor (TNF)-*α*, and vascular endothelial growth factor (VEGF), has been reported and could play very important etiological roles [2, 7, 8]. Monoclonal plasma cell proliferative disorders, such as plasma cell neoplasms and Castleman's disease, are known to be an underlying condition responsible for the syndrome [2–6]. 

 Recently, pulmonary hypertension (PH) is attracting increased attention as a clinical manifestation of the POEMS syndrome and its incidence is reported to be 5–40% [2, 3, 8]. Previous studies indicated that abnormal release of vasoactive cytokines, such as VEGF, is involved in the pathogenesis of this condition [8–12]. However, pathological studies of PH in the POEMS syndrome have been lacking.

 Herein, we report an autopsy case of a 49-year-old woman who was diagnosed with idiopathic PH and treated accordingly. The autopsy revealed the presence of smoldering plasma cell myeloma and multiple organomegaly in addition to severe PH, apparently caused by plasma cell myeloma affecting the pulmonary vasculature. From the autopsy findings, together with the clinical details, we finally concluded that the patient had suffered from PH associated with the POEMS syndrome. Pathological findings of this unique case are presented with a review of the relevant literature. 

## 2. Clinical Summary

 In 2002, a 42-year-old woman, who had been previously healthy and had no family history of PH, presented with dyspnea on exertion (New York Heart Association (NYHA) functional class II). A routine medical examination with chest X-ray and echocardiography suggested her to have PH. She underwent a right heart catheterization that revealed precapillary PH with a right atrial pressure (RAP) of 6 mmHg, a systolic pulmonary artery pressure (PAP) of 85 mmHg, a mean PAP of 51 mmHg, a pulmonary capillary wedge pressure (PCWP) of 5 mmHg, a cardiac output (CO) of 5.5 L/min, and a pulmonary vascular resistance (PVR) of 8.2 mmHg·min/L. Radiologic and echocardiographic studies as well as laboratory tests did not reveal any causes responsible for PH, such as chronic obstructive pulmonary diseases, interstitial pneumonia, congenital heart diseases, valvular heart diseases, and collagen vascular diseases. No histological examination for the lung was performed. The etiology for the PH was unclear so that she was diagnosed as having idiopathic PH and treated with epoprostenol sodium. However, her PH persisted with a systolic PAP of 70–90 mmHg. Additional medications, including sildenafil citrate and bosentan hydrate, were administered. These PH-specific therapies had been given until her death and her PH did not get worse on the 7 years of treatment. She occasionally complained of numbness of her lower extremities and difficulty in walking. Although a peripheral nerve biopsy was not performed, these symptoms were clinically diagnosed as polyneuropathy. In July 2009, she felt easily fatigued and presented with dyspnea on exertion but she was able to walk with a small baggage (NYHA functional class II). On presentation at hospital, cardiac catheterization revealed a mean RAP of 7 mmHg, a systolic PAP of 84 mmHg, a mean PAP of 50 mmHg, a PCWP of 8 mmHg, a CO of 4.1 L/min, and a PVR of 11 mmHg·min/L. These hemodynamic parameters showed no remarkable changes compared with previous findings. The patient was found to have hepatomegaly and hypoalbuminemia (serum albumin concentration; 2.1–3.3 g/dL). Laboratory data 5 months prior to her death are presented in Table 1. In contrast to the hypoalbuminemia, serum *γ*-globulin was elevated and increase in immunoglobulin (Ig) G was remarkable. Autoantibodies, including antithyroid antibodies, were detected and the level of glycosylated hemoglobin was slightly elevated. In August 2009, she presented with nausea, vomiting, and poor appetite. However, medical examination and radiological studies did not reveal any causes which could account for these symptoms. Since her poor appetite and malnutrition progressed and hypoalbuminemia persisted, she was admitted to our hospital in December 2009 and intravenous hyperalimentation therapy was administered. On her first hospital day, there was a single episode of syncope due to ventricular tachycardia. She presented with congestive heart failure and was treated with diuretics. Subsequent bronchopneumonia developed and she was treated with some antibiotics. She vomited frequently and intravascular dehydration became worse. In spite of intravascular albumin administration, the intravascular volume did not recover. Prerenal renal failure developed and cardiopulmonary function became much more disturbed. On the eighth hospital day, she eventually died of respiratory failure at the age of 49. An autopsy was performed 5 hours after her death. In the clinical course, neither hypercalcemia nor osteolytic bone lesions were detected, and plasma cells were not identified in peripheral blood. Except for the immediate antemortem prerenal renal failure, her renal function was preserved. Any cytokines such as IL-1*β*, IL-6, TNF-*α*, and VEGF were not investigated.

## 3. Pathological Findings

**Figure 1:**
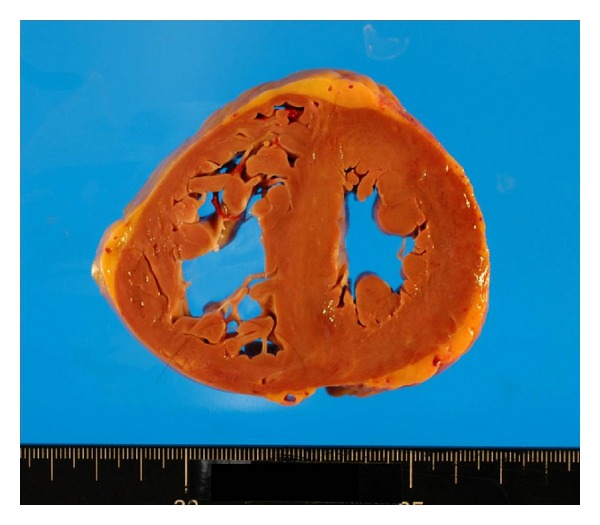
Transverse section of the heart showing marked hypertrophy of the right ventricle with a wall thickness of 7 mm. The interventricular septum is straight, giving a “D-” shaped morphology of the left ventricle, characteristic of severe PH.

**Figure 2:**
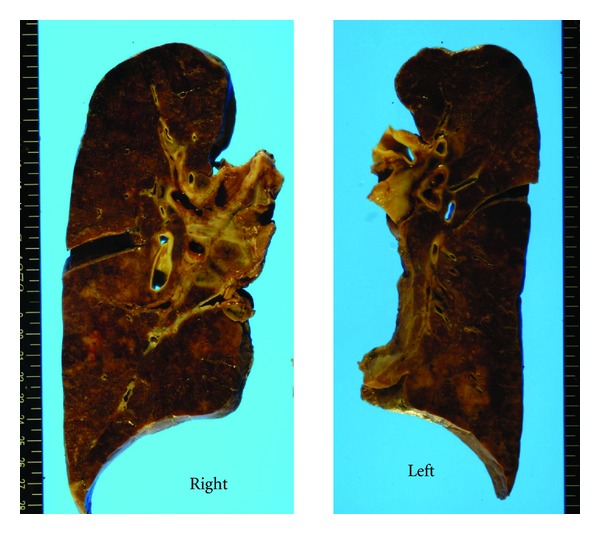
Lung cut surfaces. The left lung weighed 655 g and the right lung 890 g. Both lungs show severe congestion and hemorrhage. No tumorous lesions are grossly identified.

**Figure 3:**
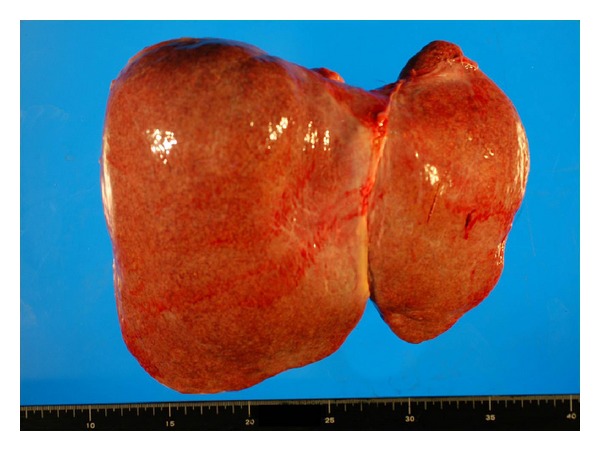
Hepatomegaly. The liver measured 27 × 22 × 9 cm in size and 2880 g in weight.

**Figure 4:**
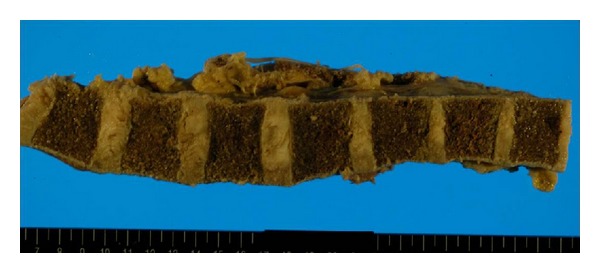
Normal gross findings of the vertebral column. Neither osteolytic nor osteosclerotic lesions are present.

 Grossly, the heart (Figure 1) weighed 500 g, with a wall thickness of 13 mm and 7 mm for the left and right ventricles, respectively. No valvular abnormalities were identified. The interventricular septum became straight to give a “D-” shaped morphology of the left ventricle. Patchy thickening of the epicardium was apparent. The pulmonary artery was free from obstructive lesions, but its wall was thickened. The aorta, renal arteries, and iliac arteries also showed wall thickening. No atherosclerotic changes were observed in any vessel. The left and right lungs, weighing 665 g and 890 g, respectively, showed severe congestion and hemorrhage (Figure 2). Marked organomegaly was observed in the liver (2880 g) (Figure 3), pancreas (400 g), and thyroid gland (60 g). The kidneys (left: 215 g, right: 190 g) were also enlarged. The spleen was slightly enlarged (165 g) with mild congestion. The alimentary tract was free from obstruction or stenosis. Marked enlargement of rugal folds (giant folds) was identified throughout the stomach. Generalized lymphadenopathy (up to 1 cm in diameter) was also found. The bones (Figure 4) appeared normal and neither osteolytic nor osteosclerotic lesions were observed. There were no vertebral deformities. No tumorous masses were identified anywhere.

**Figure 5:**
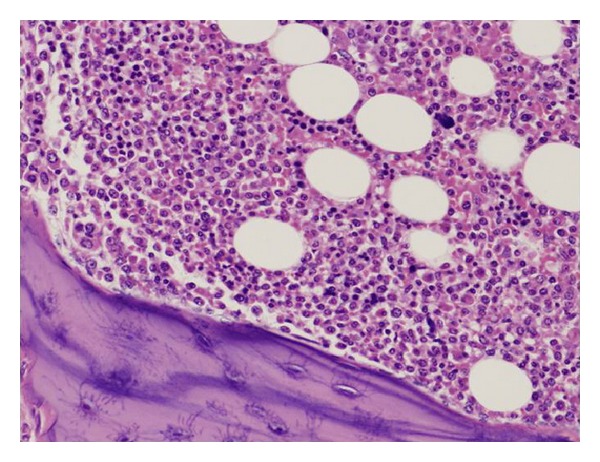
Microscopically, the lumbar vertebra shows hypercellular bone marrow with marked proliferation of plasma cells. Normal hematopoiesis appears suppressed (hematoxylin-eosin, original magnification ×200).

**Figure 6:**
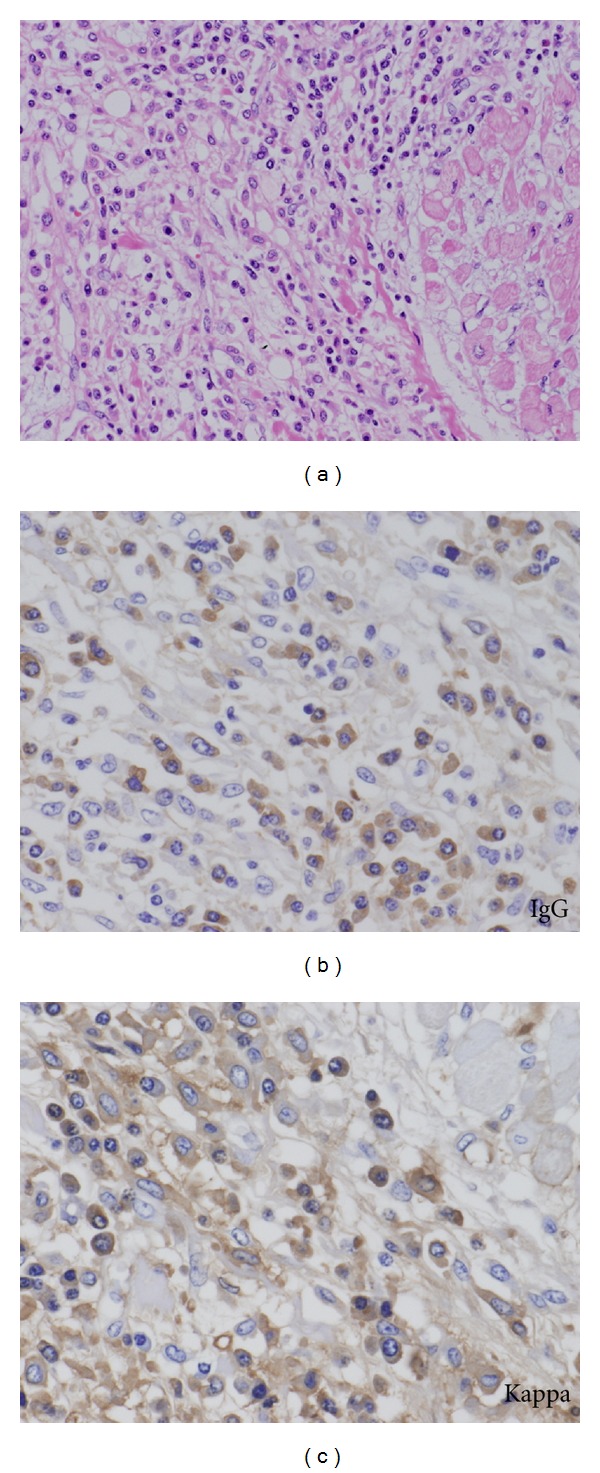
Microphotograph of the heart illustrating plasma cell invasion. Note prominent plasma cell proliferation in the region of the epicardium (a) (hematoxylin-eosin, original magnification ×200). Immunohistochemically, the plasma cells are positive for IgG (b) and kappa-light chain (c) (original magnification, ×400, resp.).

 Microscopically, prominent plasma cell proliferation was observed in many organs. The bone marrow was hypercellular with massive plasma cell proliferation, while normal hematopoiesis was suppressed (Figure 5). In the heart and large vessels, including the aorta and pulmonary artery, plasma cell proliferation was prominent, especially in the region of the epicardium and adventitia (Figure 6). Plasma cell proliferation was apparent in other organs such as the lungs, liver, pancreas, spleen, kidneys, thyroid gland, submandibular glands, alimentary tract, and lymph nodes. In the stomach and small intestine, myenteric nerve plexuses of Auerbach were involved and partially destroyed by plasma cells. In the lymph nodes, no histological findings characteristic of Castleman's disease were identified in spite of moderate plasma cell proliferation.

 Immunohistochemically, the plasma cells showed positive reactivity for IgG and kappa-light chain (Figure 6), but negative reactivity for IgA, IgD, IgE, IgM, and lambda-light chain (data not shown), which indicated monoclonal proliferation of plasma cells, that is, plasma cell myeloma. 

**Figure 7:**
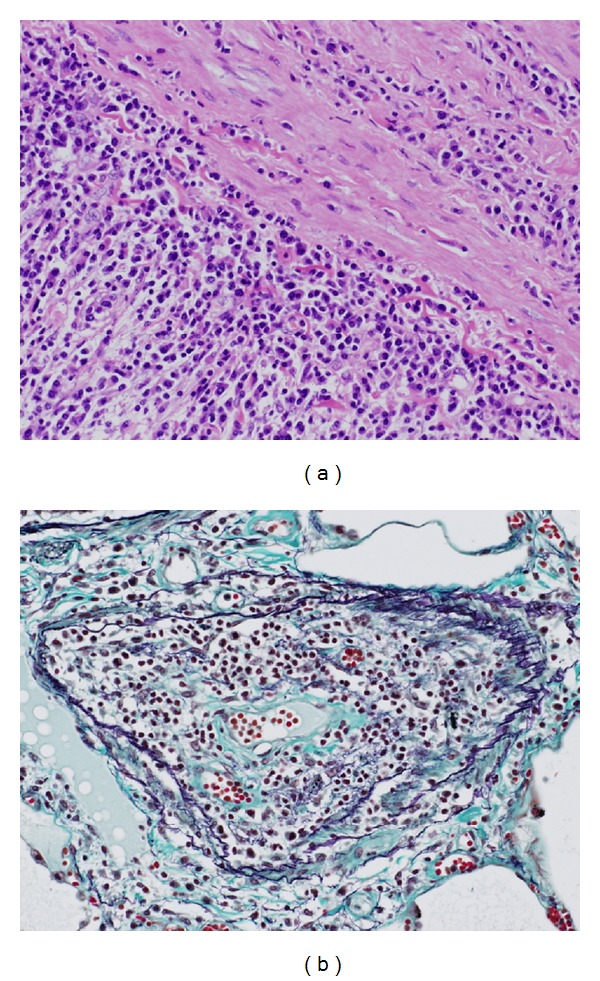
Microscopic findings of the pulmonary vasculature affected by plasma cell myeloma. (a) The pulmonary artery showing marked plasma cell proliferation. The adventitia is predominantly affected (hematoxylin-eosin, original magnification ×200). (b) A cross-section of the arteriole indicating plasma cell proliferation. The vascular wall is thickened and the lumen becomes narrow (elastica Masson, original magnification ×200).

 As for pulmonary pathology, although marked edema and hemorrhage were noted with purulent pneumonia being focally observed, the lungs were featured by prominent plasma cell invasion (Figure 7). Plasma cell proliferation was remarkable in the vascular wall, perivascular area, and interalveolar septum. There were many arteries and arterioles affected by plasma cells, showing marked wall thickening and consequent luminal stenosis. Some arterioles were occluded with mild fibrosis and recanalization being occasionally observed. There were many dilated and congestive vessels adjacent to the stenosed or occluded arterioles. There were no histological findings suggestive of idiopathic PH, such as medial hypertrophy, muscularization of small arteries, plexogenic pulmonary arteriopathy, and angiomatoid lesion [13, 14]. Amyloid deposition was not identified anywhere.

## 4. Discussion

 In the present case, prominent plasma cell proliferation was identified in many organs, especially in the cardiovascular system, shown by immunohistochemistry to be monoclonal and therefore neoplastic in nature, which leads to a pathological diagnosis of plasma cell myeloma. Although the bone marrow was affected, there were no osteolytic lesions. Also, there was no clinical evidence for end-organ damage of plasma cell myeloma. Therefore, the plasma cell myeloma was considered to be smoldering type. The present case was apparently featured by the presence of multisystem organomegaly. The highly increased serum level of IgG, together with the histopathological evidence of monoclonal proliferation of IgG-kappa-type plasma cells, implied the presence of M protein. Additionally, the presence of autoantibodies against the thyroid gland, together with impaired glucose tolerance, was regarded as laboratory evidence of endocrinopathy. The patient's symptoms such as numbness of the lower extremities and difficulty in walking were clinically concluded to be a presentation of polyneuropathy. Although we could not check peripheral nerves histologically, the fact that vertebral deformities (the most common cause of numbness and paralysis of the lower extremities) were not identified provides support for the conclusion. In the stomach and small intestine, there was plasma cell invasion in the myenteric nerve plexuses of Auerbach, which would be expected to cause bowel movement disorder and could account for some of the patient's symptoms such as nausea, vomiting, and malnutrition. This autopsy finding of plasma cell invasion in the myenteric nerve plexuses is a feature of autonomic neuropathy. Although skin changes were not apparent, based on the autopsy and clinical findings as described above, we diagnosed the patient as suffering from the POEMS syndrome [2]. The gross findings for the heart, featuring hypertrophy of the right ventricle and a “D-” shaped morphology of the left ventricle, led to an anatomical diagnosis of severe PH. Although this patient was diagnosed as having idiopathic PH and treated as such, histopathological findings of the pulmonary vasculature indicated that the cause of the PH was plasma cell myeloma. We finally concluded that the patient had suffered from PH associated with the POEMS syndrome.

 Currently, PH is becoming established as a clinical manifestation of the POEMS syndrome. Many anecdotal case reports dealing with this condition have been published [9–12, 15–24] and some studies indicate that the incidence of PH in the POEMS syndrome is 5 to 40% [2, 3, 8]. Although detailed mechanisms are unknown, some cytokines are reported to be involved in the pathogenesis [8–12, 18, 22, 25]. Rose et al. [22] found elevated serum levels of IL-6 in 2 patients with POEMS syndrome presenting with PH. Lesprit et al. [8] investigated further 5 patients with high serum concentrations of IL-1*β*, IL-6, TNF-*α*, and VEGF. Isolated increase of serum VEGF has been reported in a number of cases of POEMS syndrome presenting with PH [9, 11, 18] and the serum level of VEGF became normalized in association with amelioration of PH [11]. In our case, involvement of such cytokines in the development of PH was unclear because any such cytokines were not checked antemortem. 

 Regarding histopathological examinations on PH in POEMS syndrome patients, only a few limited studies have so far been performed [8, 18]. Lesprit et al. [8] reported histological findings in a single case, where eccentric intimal fibrosis, medial hypertrophy, and marked dilatation of the arteries and arterioles of the lungs were observed. Lewerenz et al. [18] also documented intimal fibrosis and medial hypertrophy of the pulmonary artery. These histological findings featuring plexogenic pulmonary arteriopathy mimicked the findings of idiopathic PH. In two cases of plasma cell myeloma [26, 27], neither of which satisfied the diagnostic criteria as regards the POEMS syndrome, histopathological findings of the pulmonary vasculature were demonstrated. Shiue and McNally [26] indicated that diffuse amyloid deposition in the pulmonary vessels was the cause of PH. Hattori et al. [27] reported extensive formation of plexogenic pulmonary arteries accompanied by thickening of intima and media, characteristic for idiopathic PH. In these cases mentioned above, plasma cell proliferation was not identified in the lungs. However, the present case was characterized by plasma cell proliferation in the pulmonary vasculature. Mechanical obstruction of the arterioles of the lungs with mild fibrosis due to marked plasma cell proliferation was considered to be the cause of PH. Thus, this unique histopathological feature revealed an unprecedented mechanism for PH and it provides significant clues to understanding the pathogenesis of PH. Judging from the present and previous histopathological findings, it could be concluded that the pathogenesis of PH in plasma cell disorders (PCDs) is heterogeneous. On the other hand, in most cases of the POEMS syndrome presenting with PH, plasma cells were found to almost exclusively have lambda-type light chains [2, 8, 9, 17, 18, 22, 24]. Although our case is unusual in that the light chain was of kappa type, it is congruous with the results of other studies dealing with plasma cell myeloma and PH [28, 29]. This also provides support for the conclusion that PH in PCDs has a heterogeneous pathology.

 The present case was clinically diagnosed and treated as idiopathic PH. Although the management of idiopathic PH has been improving recently, the prognosis of idiopathic PH patients remains poor [30]. In contrast, although systematic studies are lacking, many anecdotal case reports indicate that PH in PCDs may be curable with treatment by corticosteroids, immunosuppressants, antimyeloma agents, or plasmapheresis [8, 10, 12, 16, 17, 19–21, 23, 29, 31, 32]. Thus, PCD-related PH may be considered to be a particular form of PH that is potentially curable [8, 13]. The therapeutic strategies are different between idiopathic PH and PCD-related PH; therefore, establishing a differential diagnosis between them is very important. From this case in which antemortem diagnosis of POEMS syndrome could not be made, we can learn a lesson; namely, “before establishing a clinical diagnosis of idiopathic PH, it is essential for clinicians to rule out the possibility of the PCD-related PH.” For this purpose, it is indispensable to perform serum protein electrophoresis for detection of M protein, to estimate cytokine markers such as VEGF, and to radiologically evaluate spinal bones. A histopathological examination with lung tissue should be aggressively employed if indicated. 

## 5. Conclusion

 We report an autopsy case of POEMS syndrome presenting with severe PH caused by smoldering plasma cell myeloma. This case showed a unique pulmonary vascular pathology featuring plasma cell proliferation and provides clues to understanding the mechanism of PH in PCDs, a condition whose pathogenesis should be considered heterogeneous. Therefore, in order to better understand PH in the PCDs, much more pathogenetic analyses, especially with histopathological methods, should be conducted [13].

## Figures and Tables

**Table 1:** Laboratory data of the patient.

Leukocyte	6900/*μ*L	(3500–9000)
Hemoglobin	11.3 g/dL	(11.5–15.5)
Hematocrit	34.8%	(34.0–46.0)
Platelet	68000/*μ*L	(150000–380000)
Blood urea nitrogen	7.1 mg/dL	(8.0–20.0)
Creatinine	0.5 mg/dL	(0.2–0.8)
Glycosylated hemoglobin	6.6%	(4.3–5.8)
Total protein	8.0 g/dL	(6.5–8.0)
Albumin	3.3 g/dL	(4.0–5.4)
*α*1-Globulin	0.3 g/dL	(0.1-0.2)
*α*2-Globulin	0.8 g/dL	(0.4–0.7)
*β*-Globulin	0.7 g/dL	(0.4–0.8)
*γ*-Globulin	3.0 g/dL	(0.7–1.6)
Immunoglobulin G (IgG)	3150 mg/dL	(778–1794)
Immunoglobulin A (IgA)	245 mg/dL	(80–413)
Immunoglobulin M (IgM)	64 mg/dL	(37–254)
Free T4	1.7 ng/dL	(0.7–1.5)
Free T3	2.1 pg/mL	(1.6–3.2)
Thyroid stimulating hormone	1.9 *μ*IU/mL	(0.4–5.2)
Antithyroid peroxidase antibody	6.1 U/mL	(0.0–0.3)
Antithyroglobulin antibody	<0.3 U/mL	(0.0–0.3)
Thyroid stimulating hormone receptor antibody	33%	(0–15)

Parentheses indicate normal range.
